# Sunitinib-induced severe toxicities in a Japanese patient with the *ABCG2* 421 AA genotype

**DOI:** 10.1186/1471-2407-14-964

**Published:** 2014-12-16

**Authors:** Yuji Miura, Chiyo K Imamura, Koya Fukunaga, Yoshihiko Katsuyama, Koichi Suyama, Toshikazu Okaneya, Taisei Mushiroda, Yuichi Ando, Toshimi Takano, Yusuke Tanigawara

**Affiliations:** Department of Medical Oncology, Toranomon Hospital, 2-2-2 Toranomon, Minato-ku, Tokyo, 105-8470 Japan; Department of Clinical Pharmacokinetics and Pharmacodynamics, School of Medicine, Keio University, 35 Shinanomachi, Shinjuku-ku, Tokyo, 160-8582 Japan; Laboratory for Pharmacogenomics, RIKEN Center for Integrative Medical Sciences, 1-7-22 Suehirocho, Tsurumi-ku, Yokohama, Kanagawa, 230-0045 Japan; Department of Pharmacy, Shinshu University Hospital, 3-1-1 Asahi, Matsumoto, Nagano, 390-8621 Japan; Department of Urology, Toranomon Hospital, 2-2-2 Toranomon, Minato-ku, Tokyo, 105-8470 Japan; Department of Clinical Oncology and Chemotherapy, Nagoya University Hospital, 65 Tsurumai-cho, Showa-ku, Nagoya, Aichi, 466-8560 Japan

**Keywords:** Renal cell carcinoma, Sunitinib, Pharmacokinetics, Pharmacogenetics, Single-nucleotide polymorphism, ABCG2

## Abstract

**Background:**

Sunitinib is a multi-targeted receptor tyrosine kinase inhibitor that acts against receptors for vascular endothelial growth factor and platelet-derived growth factor. Common toxicities of sunitinib treatment include hypertension, hand–foot syndrome, vomiting, and diarrhea, and the proportion of grade 3 or 4 adverse events relating to sunitinib treatment range from 1 to 13% for all categories. It is reported that increased exposure to sunitinib is associated with improved clinical outcomes but also carries an increased risk of adverse effects.

**Case presentation:**

A 73-year-old Japanese woman with metastatic renal cell carcinoma who received sunitinib at a dose of 50 mg once daily suffered a high-grade fever on day 11 of treatment. Sunitinib treatment was discontinued on day 12; however, severe thrombocytopenia and transaminase elevation occurred and persisted more than a week. Additionally, severe hypoxia due to pleural effusion and pulmonary edema developed despite immediate discontinuation of sunitinib. On day 14, three days after the discontinuation of sunitinib, the plasma concentrations of sunitinib and its major active metabolite N-desethyl sunitinib (SU12662) were extremely high (131.9 ng/mL and 28.4 ng/mL, respectively). By day 25, all toxicities had resolved, and a CT scan revealed marked tumor shrinkage. Genotyping of seven single-nucleotide polymorphisms that are potentially relevant to the pharmacokinetics of sunitinib was performed. The patient’s genotype of *ABCG2* (ATP-binding cassette, sub-family G (WHITE), member 2) 421C > A was homozygous for the variant allele (AA), which was reported to be associated with high exposure to sunitinib. Therefore, we speculated that the extremely high plasma concentrations of sunitinib and SU12662 caused by the *ABCG2* 421 AA genotype might have resulted in severe toxicities to the patient.

**Conclusion:**

The minor allele frequencies of *ABCG2* 421C > A are approximately three-fold higher in Asians than in Caucasians. Our report suggests that pharmacogenetic factors should be considered when severe and rapid-onset adverse drug reactions occur in Asian patients, including Japanese treated with sunitinib.

**Electronic supplementary material:**

The online version of this article (doi:10.1186/1471-2407-14-964) contains supplementary material, which is available to authorized users.

## Background

Sunitinib is a multi-targeted receptor tyrosine kinase inhibitor that acts against receptors of vascular endothelial growth factor (VEGF) and platelet-derived growth factor [1]. Sunitinib therapy has dramatically improved the clinical outcome of patients with advanced renal cell carcinoma (RCC) [2]. Common toxicities with sunitinib treatment include hypertension, hand–foot syndrome, vomiting, and diarrhea, and the proportion of grade 3 or 4 adverse events associated with sunitinib range from 1 to 13% for all categories [2]. A pharmacokinetics/pharmacodynamics meta-analysis in patients with advanced solid tumors, including patients with gastrointestinal stromal tumor (GIST) and metastatic RCC, indicated that increased exposure to sunitinib was associated with improved clinical outcomes in parallel with a degree of increased risk of adverse effects [3]. Previous studies suggested that several single-nucleotide polymorphisms (SNPs), including those in genes that are relevant to the pharmacokinetics of sunitinib such as *CYP3A5* (cytochrome P450, family 3, subfamily A, polypeptide 5) and *ABCG2* (ATP-binding cassette, sub-family G (WHITE), member 2), might be associated with its toxicities [4, 5].

## Case presentation

**Figure 1:**
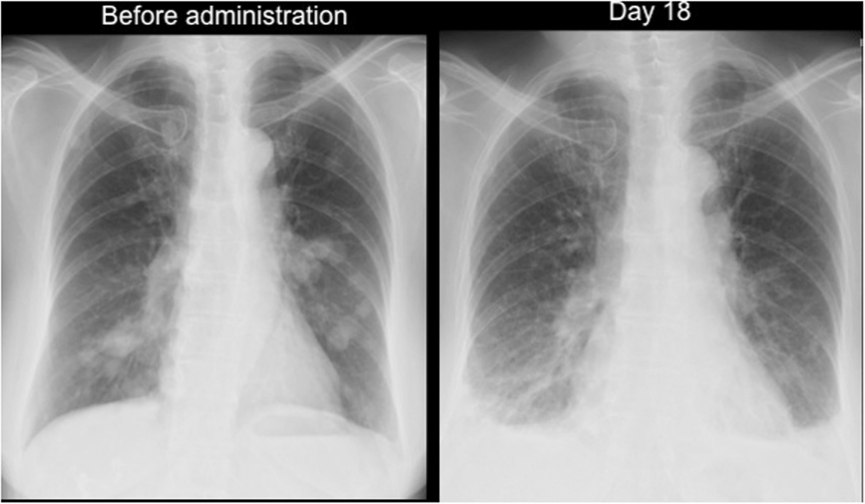
**Chest X-ray images.** Chest X-ray images before and on day 18 of sunitinib administration revealed bilateral multiple lung nodules and bilateral pleural effusion, respectively.

**Figure 2:**
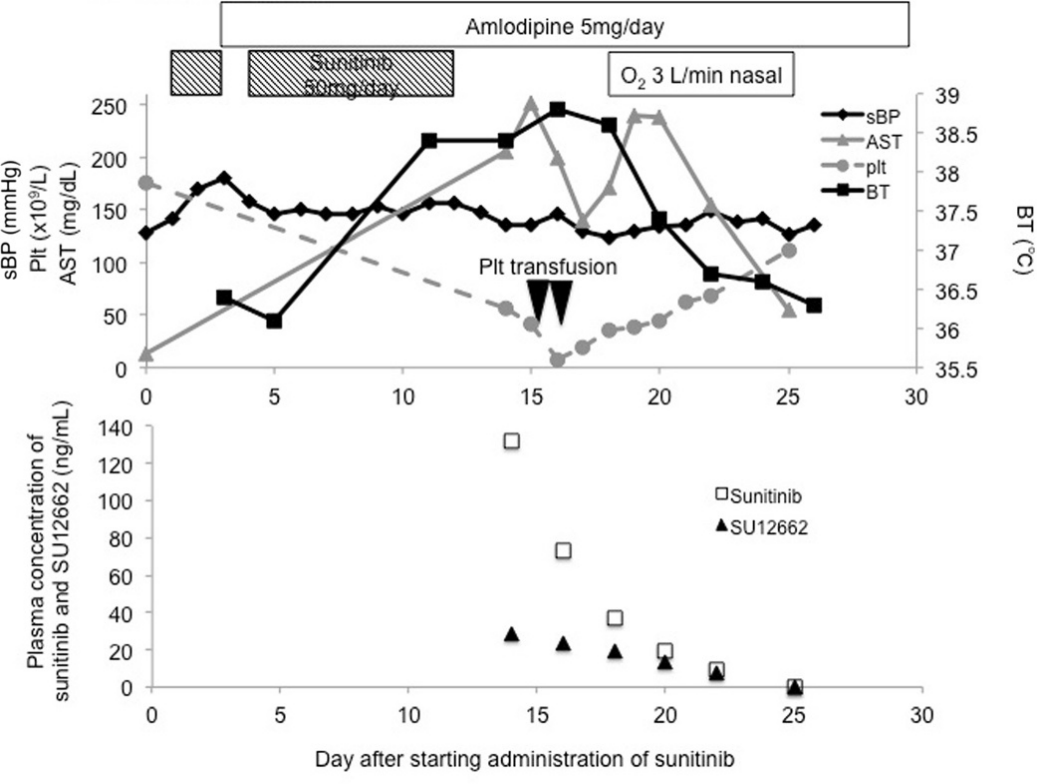
**Clinical courses.** Clinical courses including vital signs, laboratory data, and treatment (upper). Plasma concentrations of sunitinib and SU12662 (lower). Each arrowhead of Plt transfusion represents the transfusion of 10 units of platelets. sBP, systolic blood pressure; BT, body temperature; AST, aspartate aminotransferase; Plt, platelets.

In June 2011, a previously healthy 73-year-old female was found to have multiple lung nodules via a chest X-ray performed as part of a routine health checkup. A computer tomography (CT) scan revealed a 6-cm-sized left kidney mass and multiple lung nodules. She had no past medical history, such as hypertension, cardiovascular disease, or hyperlipidemia; with the exception that she had received orally-dosed alendronate at 35 mg a week for the prevention of osteoporosis. The patient was referred to our hospital and underwent a left-side nephrectomy. Histopathologically, the left kidney mass was diagnosed as a clear cell RCC with sarcomatoid components, a Fuhrman nuclear grade of 4, and pT3a. Four weeks after the nephrectomy, sunitinib therapy was initiated at a standard regimen of 50 mg once daily for 4 weeks, followed by 2 weeks without treatment. Physical examination of the patient before starting treatment revealed no abnormalities: her blood pressure was 128/76 mmHg, SpO_2_ was 97% in room air, and performance status was 0. Blood examination showed normal complete blood counts, creatinine, transaminases, and bilirubin. On day 3 of sunitinib administration, the patient’s blood pressure increased to 180/90 mmHg. Treatment with amlodipine (5 mg, once daily) was commenced, and the sunitinib treatment was temporarily discontinued. On day 4, the patient’s hypertension had improved and sunitinib treatment was resumed at same dose. On day 11, the patient suffered from a fever of 38.4°C; the sunitinib treatment was discontinued on day 12. On day 14, she was admitted to hospital because the fever had persisted and she developed fatigue, anorexia, dyspnea and hypoxia (SpO_2_ 93% room air), all of which were of grade 2 according to the Common Terminology Criteria for Adverse Events (CTCAE), version 4.03. A chest X-ray did not reveal any signs of pleural effusion or heart congestion. The laboratory value showed thrombocytopenia (platelet count, 56 × 10^9^/L) and liver dysfunction (aspartate aminotransferase [AST], 205 IU/L; alanine aminotransferase [ALT], 151 IU/L). We ruled out infectious disease and made a presumed diagnosis of sunitinib-related toxicities, because no evidence of infection was detected in blood, urine, and sputum cultures. On day 15 and 16, platelet transfusions were performed. On day 18, the patient’s dyspnea and hypoxia were exacerbated to grade 3 (SpO_2_ was 88% room air at rest, and continuous supplemental oxygen was required at rest). A chest X-ray and CT scan revealed bilateral pleural effusion and pulmonary edema (Figure 1). An echocardiography showed no signs of cardiac dysfunction. Supportive care using supplemental oxygen and diuretics were undertaken. On day 25, all toxicities including fever, dyspnea, hypoxia, liver dysfunction, and thrombocytopenia had resolved (Figure 2, upper).

**Figure 3:**
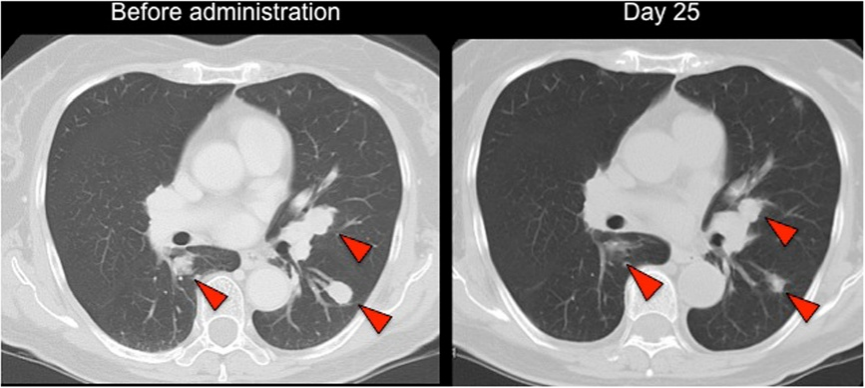
**CT images.** Computer tomography images before and on day 15 of sunitinib administration revealed that the size of the lung metastases was remarkably decreased by sunitinib treatment. Each arrowhead represents lung metastases.

The patient underwent an objective response evaluation based on a CT scan on day 25. The tumor size had decreased to 30% based on the Response Evaluation Criteria in Solid Tumors (RECIST), version 1.1, despite the administration of sunitinib for just 10 days (Figure 3).

For subsequent elucidation and evaluation of the case, the plasma concentrations of sunitinib and its major metabolite N-desethyl sunitinib (SU12662), which has equal activity to sunitinib, from the plasma samples stored at −20°C on days 14, 16, 18, 20, 22, and 25 were determined by high performance liquid chromatography (HPLC)-UV. HPLC-UV was equipped with a chromatographic system consisted of a model L-7100 pump, D-7000 interface, L-7400 UV-visible detector, L-7200 autosampler and L-7300 column oven (Hitachi, Japan). Chromatographic separations were obtained under gradient conditions using a CAPCELL PAK C18 MG analytical column (250 × 2.0 mm, 5 μm particle size; Shiseido, Japan). Standard curves were prepared in the concentration range of 5 to 4mL/mL for sunitinib and 5 to 2mL/mL for SU12662 [6]. The plasma concentrations of sunitinib and SU12662 on day 14 were 131.9 ng/mL and 28.4 ng/mL, respectively (Figure 2, lower).

Genotyping of seven SNPs in *CYP3A5* (6986A > G), *ABCB1* (ATP-binding cassette, sub-family B (MDR/TAP), member 1; 1236C > T, 2677G > T/A, and 3435C > T), and *ABCG2* (34G > A, 376G > A, and 421C > A) that are potentially relevant to the pharmacokinetics of sunitinib [7–11] was performed using a genomic DNA sample extracted from formalin-fixed paraffin-embedded normal renal tissue blocks with a QIAamp DNA FFPE Tissue Kit (QIAGEN, Germany), because blood was not available for DNA extraction owing to this being a posthumous investigation. The institutional ethics committees of Toranomon Hospital and RIKEN approved this study. Written informed consent was obtained from the patient’s representatives. PCR amplification was first carried out in a 20-μl reaction mixture containing 10 ng of genomic DNA, 10 × Vogelstein buffer, 20 mM dNTPs, 2.5 U of TAKARA Ex Taq (Takara Bio, Otsu, Japan) and 10 pmol of each forward and reverse primer (Additional file 1: Table S1) under the following conditions using a GeneAmp PCR System 9700 (Applied Biosystems, Foster City, CA, USA): initial denaturation at 95°C for 5 min, 40 cycles at 95°C for 15 sec, 60°C for 30 sec and 72°C for 90 sec, and a final extension at 72°C for 5 min. Direct sequencing of the PCR products was then performed using the BigDye Terminator v.3.1 Cycle Sequencing Kit according to the manufacturer’s protocol on an ABI 3730xl DNA Analyzer (Applied Biosystems). The SNPs of *CYP3A5*, *ABCB1*, and *ABCG2,* which potentially affect sunitinib metabolism and transport, were genotyped. The genotypes of four SNPs in *CYP3A5* and *ABCB1* were heterozygous for the variant alleles in this patient. The genotypes of *ABCG2* 34G > A, 376G > A, and 421C > A were homozygous for the wild-type, wild-type, and variant alleles, respectively (Table 1).

**Table 1:** **Genotypes of seven SNPs in** ***CYP3A5*** **,** ***ABCB1*** **and** ***ABCG2***

Gene	SNP	Allele	Amino acid	Genotype
*CYP3A5*	rs776746	6986A > G	Splice Site	AG
*ABCB1*	rs1128503	1236C > T	G412G	CT
*ABCB1*	rs2032582	2677G > T/A	A893S/T	GT
*ABCB1*	rs1045642	3435C > T	I1145I	CT
*ABCG2*	rs2231137	34G > A	V12M	GG
*ABCG2*	rs72552713	376G > A	Q126X	GG
*ABCG2*	rs2231142	421C > A	Q141K	AA

## Conclusions

We here report a patient with metastatic RCC who developed severe sunitinib-induced toxicities, including hypertension, high fever, liver dysfunction, and thrombocytopenia, after only 10 days administration of sunitinib. Despite immediate discontinuation of sunitinib, these symptoms persisted for over a week, and subsequent life-threatening hypoxia due to pleural effusion and pulmonary edema occurred. A phase I dose-escalation study of sunitinib administered daily on a 4-weeks-on, 2-weeks-off schedule indicated that most patients with a dose-limiting toxicity (DLT), including thrombocytopenia, hypertension and asthenia, had a combined (sunitinib plus SU012662) trough plasma concentration of ≥1mL/mL [12]. The combined plasma concentration of sunitinib and SU12662 on day 14 in this case was 160.3 ng/mL, even when measured at 3 days after the discontinuation of sunitinib.

Several factors can be attributed to the pathogenesis of the significantly high plasma concentrations of sunitinib and SU12662. First, co-administration of sunitinib with strong inhibitors of *CYP3A4* and/or intake of grapefruit increase sunitinib concentration; however, our patient did not take these in parallel with sunitinib treatment. Second, we assumed that the patient might be a carrier of the variant alleles of SNPs in *CYP3A5*, *ABCB1*, and/or *ABCG2* that cause higher plasma concentrations of sunitinib. *ABCG2*, the so-called breast cancer resistance protein (BCRP), is an ATP-binding cassette (ABC) drug transporter that facilitates the efflux of various tyrosine kinase inhibitors, including sunitinib, from cells. The *ABCG2* 421C > A polymorphism is associated with reduced ABCG2 protein expression and/or activity [13]. The reduced protein levels of the AA genotype in *ABCG2* 421C > A in the apical membranes of small intestinal enterocytes, hepatocytes, and renal proximal tubule epithelial cells might affect intestinal absorption and/or elimination after oral administration of sunitinib, leading to extremely high systemic exposure to sunitinib. Kim et al. reported that the *ABCG2* 421 AA genotype is mostly associated with the risk of sunitinib-related toxicities, such as thrombocytopenia, neutropenia and hand–foot syndrome, in Korean patients with metastatic RCC; however, the pharmacokinetics data was not shown [5]. Mizuno et al. reported a Japanese case with the *ABCG2* 421 AA genotype that developed severe toxicities due to high exposure of sunitinib and SU12662 [14]. Additionally, they demonstrated the *ABCG2* 421A > C genotype as a significant covariate associated with lower oral clearance by generating a population pharmacokinetics model of sunitinib from 19 Japanese patients with RCC, in which the number of patients with *ABCG2* 421 CC, CA and AA genotypes were 10, 8, and 1, respectively. The plasma concentration–time profiles simulated with repeated sunitinib doses of 50 mg/day based on the model showed that trough concentrations after 14 days for most *ABCG2* 421C > A carriers exceed 100–130 ng/mL [15]. However, the plasma concentration of sunitinib in our patient on day 14, when the administration of sunitinib has been discontinued for three days after repeated doses of 50 mg/day, was 131.9 ng/mL.

The elimination half-life of sunitinib calculated from its plasma concentrations on days 14, 16, 18, 20, and 22 in our patient was 50.1 hours, similar to that previously reported (41–86 hours) [12]. Therefore, it could be explained that the high exposure of sunitinib was caused by the loss of ABCG2 protein expression by genetic polymorphism. In the present study, the genotypes of SNPs in *CYP3A5* and *ABCB1*, which are potentially related to the pharmacokinetics of sunitinib [7–11], were heterozygous for the variant alleles, indicating that these SNPs might also be involved in the high systemic exposure of sunitinib to the patient.

Another population pharmacokinetics study also identified ethnic background as a significant covariate for the prediction of oral clearance [16]. The frequency of the *ABCG2* 421 AA genotype is higher in Asian populations (Japanese, 7% [13]; Korean, 8% [17]; and Chinese, 12% [18]) than in non-Asian populations (Caucasian, 1.7% and African, 0.2% [18]). Thus, this racial difference in the frequency of the *ABCG2* 421 AA genotype could explain the higher frequency of grade 3 and 4 sunitinib-related toxicities in Asians than in non-Asians [19, 20].

Our report suggests that pharmacogenetic factors should be considered when severe and rapid-onset adverse drug reactions develop in Asians treated with sunitinib.

### Consent

Written informed consent was obtained from the next of kin of the patient for the publication of this case report and any accompanying images.

## Electronic supplementary material

Additional file 1: Table S1: Primer sequences for PCR and direct sequencing. (DOC 33 KB)

## Abbreviations

VEGFR: Vascular endothelial growth factor receptor
RCC: Renal cell carcinoma
GIST: Gastrointestinal stromal tumor
CT: Computer tomography
CTCAE: Common terminology criteria for adverse events
AST: Aspartate aminotransferase
ALT: Alanine aminotransferase
RECIST: Response evaluation criteria in Solid tumors
HPLC: High performance liquid chromatography
SNPs: Single-nucleotide polymorphisms
DLT: dose-limiting toxicity
BCRP: Breast cancer resistance protein
ABC: ATP-binding cassette.
