# Supplementary material for: Sunitinib-induced severe toxicities in a Japanese patient with the ABCG2 421 AA genotype
Source: BMC Cancer. 2014 Dec 16;14:964. doi: 10.1186/1471-2407-14-964 (PMC4301945; doi:10.1186/1471-2407-14-964)
Supplement: Supplementary file 1 — Additional file 1: Table S1: Primer sequences for PCR and direct sequencing. (DOC 33 KB) [file 12885_2014_5108_MOESM1_ESM.doc]

**Additional file 1: Table S1** Primer sequences for PCR and direct sequencing.

| **Gene** | **SNP** | **Forward (5’-3’)** | **Reverse (5’-3’)** | **Product size (bp)** |
| --- | --- | --- | --- | --- |
| CYP3A5 | rs776746 | ACCACCCAGCTTAACGAATG | TACGTTCTGTGTGGGGACAA | 315 |
| ABCB1 | rs1128503 | AGAGTGGGCACAAACCAGAT | ACTGTTGTGCTCTTCCCACA | 343 |
| ABCB1 | rs2032582 | GTCCAAGAACTGGCTTTGCT | TGTTGTCTGGACAAGCACTG | 239 |
| ABCB1 | rs1045642 | TTCAAAGTGTGCTGGTCCTG | CATGCTCCCAGGCTGTTTAT | 291 |
| ABCG2 | rs2231137 | TGCCTTCAGGTCATTGGAAG | TGCCTGTCTTCCCATTTAGG | 291 |
| ABCG2 | rs72552713 | CCAGATTCTCCCTGCCTTTT | GCTGCAAGGAAAGATCCAAG | 278 |
| ABCG2 | rs2231142 | CCACACAGGGAAAGTCCTAC | GTTGTGATGGGCACTCTGAC | 205 |
